# 

**Published:** 1891-03

**Authors:** 


Bristol Medico-Chirurgical Journal.

MARCH, 1891.

CONTENTS.

| | |
|---|---|
| Original Papers: | Page |
| Puerperal Eclampsia. By J. G. Swayne, M.D. ... ... ... ... .. ... | 1 |
| Case of Poisoning by Rhus Venenata. By Arthur W. Prichard, M.R.C.S. ... ... ... ... ... ... ... ... ... ... ... ... ... | 22 |
| A Case of Laryngeal Tuberculosis. By P. Watson Williams, M.B. Lond. ... ... ... ... ... . ... ... ... ... ... ... ... ... | 27 |
| Progress of the Medical Sciences ... ... ... ... ... ... ... ... | 31 |
| Reviews of Books ... ... ... ... ... ... ... ... ... ... ... ... ... | 50 |
| Notes on Preparations for the Sick ... ... ... ... ... ... ... ... | 61 |
| Meetings ... ... ... ... ... ... ... ... ... ... ... ... ... ... ... | 63 |
| Library .. ... ... ... ... ... ... ... ... ... ... ... ... ... ... | 66 |
| Obituary ... ... ... ... ... ... ... ... ... ... ... ... ... ... ... | 67 |
| Royal College of Physicians of Edinburgh ... ... ... ... ... ... | 70 |
| Scraps ... ... ... ... ... ... ... ... ... ... ... ... ... ... ... ... | 71 |

